# Inflammatory bowel disease activity assessed by fecal calprotectin and lactoferrin: correlation with laboratory parameters, clinical, endoscopic and histological indexes

**DOI:** 10.1186/1756-0500-2-221

**Published:** 2009-10-29

**Authors:** Andrea Vieira, Chia Bin Fang, Ernani Geraldo Rolim, Wilmar Artur Klug, Flávio Steinwurz, Lucio Giovanni Battista Rossini, Paulo Azevedo Candelária

**Affiliations:** 1 Clinic of Gastroenterology, Department of Medicine of the Irmandade de Misericórdia da Santa Casa de São Paulo, São Paulo, Brazil; 2 Service of Coloproctology, Department of Surgery of the Irmandade de Misericórdia da Santa Casa de São Paulo, São Paulo, Brazil; 3 Hospital Israelita Albert Einstein, São Paulo, Brazil; 4 Service of Endoscopy of Irmandade Santa Casa de São Paulo, São Paulo, Brasil

## Abstract

**Background:**

Research has shown that fecal biomarkers are useful to assess the activity of inflammatory bowel disease (IBD). The aim of the study is: to evaluate the efficacy of the fecal lactoferrin and calprotectin as indicators of inflammatory activity.

**Findings:**

A total of 78 patients presenting inflammatory bowel disease were evaluated. Blood tests, the Crohn's Disease Activity Index (CDAI), Mayo Disease Activity Index (MDAI), and Crohn's Disease Endoscopic Index of Severity (CDEIS) were used for the clinical and endoscopic evaluation. Two tests were performed on the fecal samples, to check the levels of calprotectin and lactoferrin. The performance of these fecal markers for detection of inflammation with reference to endoscopic and histological inflammatory activity was assessed and calculated sensitivity, specificity, accuracy.

A total of 52 patient's samples whose histological evaluations showed inflammation, 49 were lactoferrin-positive, and 40 were calprotectin-positive (p = 0.000). Lactoferrin and calprotectin findings correlated with C-reactive protein in both the CD and UC groups (p = 0.006; p = 0.000), with CDAI values (p = 0.043; 0.010), CDEIS values in DC cases (p = 0,000; 0.000), and with MDAI values in UC cases (p = 0.000).

**Conclusion:**

Fecal lactoferrin and calprotectin are highly sensitive and specific markers for detecting intestinal inflammation. Levels of fecal calprotectin have a proportional correlation to the degree of inflammation of the intestinal mucosa.

## Findings

Inflammatory Bowel Disease (IBD) includes Crohn's Disease (CD) and Ulcerative Colitis (UC). These are chronic idiopathic conditions, marked by recurrent episodes of inflammation of the gastrointestinal tract, interspersed with periods of remission. In order to determine the degree of inflammatory activity, it is of the utmost importance to monitor patient's clinical evolution and adjust their therapy [1].

Various indexes are used to evaluate the activity of the disease, which differ from each other in terms of being more subjective (clinical), more objective (endoscopic-histological) or a combination of the two. However, despite the different indexes available, there is not yet any consensus in the literature as to which is the most valid. Laboratory parameters such as C-reactive protein (CRP), erythrocyte sedimentation rate (ESR), and hemoglobin, among others, are not specific to active IBD, which makes it difficult to use them routinely as markers of inflammatory activity in clinical practice [2].

Some authors consider a colonoscopy with biopsy to be the best means for evaluating inflammation location, extent, and severity; aside from being an invasive method, this approach carries risks of complications [3]. Various studies have described fecal markers as powerful biomarkers of inflammation of the intestinal mucosa in patients with IBD [3-8]. Fecal markers selected and studied as indicators of inflammation include neutrophil granule proteins, lactoferrin and calprotectin [2-8].

Lactoferrin is an iron-containing glycoprotein secreted by the majority of mucosal membranes. It is the main component of secondary polymorphonuclear granules, which are the prime cells of an acute inflammatory response. Other hematopoietic cells, such as monocytes and lymphocytes, do not contain lactoferrin. In intestinal inflammation, leukocytes invade the mucosa, which results in an increase in the excretion of lactoferrin into the feces [5,7]. Calprotectin is a calcium-containing protein that makes up 5% of the total protein and 60% of the cytosolic protein of neutrophil. It has bacteriostatic and fungistatic properties and is found in feces at levels six times higher than that in plasma [[1,6], and [8]].

Several studies have compared fecal lactoferrin and calprotectin with activity indexes and/or endoscopic/histological evaluation to verify intestinal inflammation in IBD patients. The results of these studies are promising, having demonstrated that these markers are useful in detecting inflammation and differentiating it from other diseases as well as in predicting recurrence for periods of up to one year [[1,2,5,8], and [9]]. Hence the present study sought to evaluate the efficacy of fecal excretion biomarkers. Specifically, the first aim was to assess fecal lactoferrin and calprotectin as indicators of IBD activity by determining how well these indicators correlate with other indexes of inflammatory activity including laboratory measures and endoscopic and histological evaluation.

## Patients and Methods

### Patients

A total of 78 patients, 38 with CD and 40 with UC, were recruited for this study, in accordance with the following criteria for inclusion: (1) age 18 years or older; (2) written informed consent given prior to participation such that the terms were clear and free consent, as approved by the Research Ethics Committee of the Central Hospital of the Santa Casa of São Paulo; (3) being willing and available to undertake all the procedures of the study, such as collection of stool and blood samples and undergoing a colonoscopy and/or double-balloon enteroscopy; and (4) being able to come to the clinic where the study was taking place, whenever necessary. There were five exclusion criteria as follows: (1) testing positive for HIV or Hepatitis B or C; (2) history of infectious diarrhea during the previous six months; (3) infection with intestinal parasites; (4) colostomy or ileostomy up to one month before study enrollment; and (5) prior diagnosis with intestinal cancer. Clinical data for the study's population are summarized in Table 1.

**Table 1:** Summary of IBD patients' clinical data

Illness	CD	UC
Number of patients	38	40
Sex (male/female)	24/14	21/19
Median age (minimum-maximum)	37 (18-64)	46 (19-80)
Ethnicity (white, asian, mixed-race, black	32/0/5/1	33/1/3/3
Family history of IBD (yes/no)	4/34	6/34
Extent of disease		
Terminal ileum	12	
Ileum and colon	13	
Pancolitis	11	12
Rectal + sigmoid + descending colon		3
Rectal	2	25
Surgery intended (Yes/no)	4/34	3/37
Treatment		
Aminosalicylate	14	28
Immunosuppressant	16	11
Anti-TNF	8	1

## Methods

### The recruitment of patients

The patients were subjected to blood tests (hemoglobin, hematocrit, leukocytes, platelets, CRP, ESR) and parasitological tests on their feces. The patients selected were advised that they should return to the clinic with two samples of fresh stool collected on the day of or, at most, 24 hours before their visit. In addition, they were asked about their symptoms over the preceding seven days in the case of patients with CD, and over the last three days in the case of patients with UC, thereby allowing for the calculation of the Crohn's Disease Activity Index (CDAI) and the Mayo Disease Activity Index (MDAI). CDAI and MDAI values compatible with disease activity were > 150 points, and > 0, respectively.

### Fecal tests

During the study, patients' stool samples were subjected to two tests: a quantitative one to determine the level of calprotectin and a qualitative one to identify the presence of lactoferrin. Quantitative measurement of calprotectin was performed using PhiCal™ Test (Calpro AS, Oslo, Norway). One to 5 g stool were collected and stored at -20°C. Specimens were incubated with polyclonal rabbit antibodies against calprotectin. Bound calprotectin was allowed to reach with alkaline phosphatase labeled, imunoaffinity purified IgG (rabbit) antibodies against calprotectin. After adding enzyme's substrate O.D. values were read in an Elisa reader and compared to negative and positive kit controls. All steps were carried out following manufactures' instructions.

Qualitative measurement of elevated levels of lactoferrin was performed using IBD-CHEK test (Techlab, Blacksburg, VA, USA). One to 5 g stool were collected. Specimens were incubated with immobilized polyclonal antibody against lactoferrin and then detected with polyclonal antibody conjugated to horseradish peroxidase. After substrate addition, colored enzyme-antibody-antigen complexes formed in the presence of lactoferrin were measured in an Elisa reader and compared to negative and positive kit controls. All steps were carried out following manufactures' instructions.

### Endoscopic examinations

Patients with UC were given a colonoscopy, while those with CD were given a double-balloon enteroscopy (was performed through the rectum) to access inflammatory activity. Two endoscopists (always the same ones) did all of the analyses by video during the procedures without knowledge of the results of the patients' blood tests or fecal tests. Mayo Disease Activity Index (endoscopic sub score), and Crohn's Disease Endoscopic Index of Severity (values >/=3 were considered positive for intestinal inflammation) were used for endoscopic evaluation. Biopsies were performed each 10 cm in normal or pathological areas of bowel.

### Histological evaluation

The following data were assessed by histology: the presence of neutrophils in the lamina propria, the presence of erosion and/or ulceration, and crypt aggression.

### Statistical analysis

The statistical program SPSS (Statistical Package for Social Sciences for Windows version 17.0) was used to analyze the data, with the 5% level of significance (0.05). Spearman's Rank Correlation was applied to evaluate all the variables studied, namely levels of hemoglobin, hematocrit, leukocytes, platelets, C-reactive protein, hemosedimentation rates, fecal lactoferrin and calprotectin, CDAI, MDAI, CDEIS, and histological evaluation. Sensitivity of the variables was also studied in terms of their positive predictive value, negative predictive value, and accuracy relative to that of endoscopic and histological evaluation.

The best calprotectin cut-off point was calculated using the area under the ROC (receiver operating characteristic) curve.

## Results

### Fecal tests

**Figure 1:**
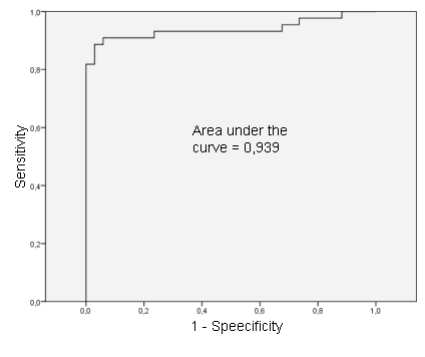
**Area under the receiver operating characteristic (ROC) curve to analyze the best cut off of calprotectin**.

**Figure 2:**
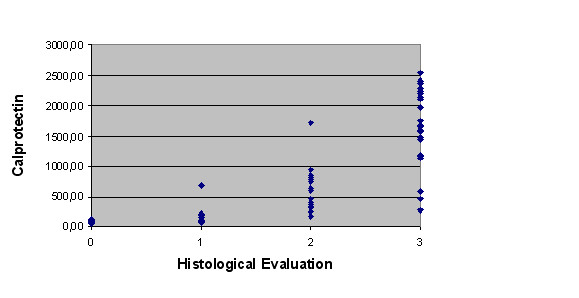
**Comparison between fecal Calprotectin and intensity of histological inflammation**. P=0.000 (Spearman Rank Correlation). Histological Evaluation (degree of inflammation):  0: absent, 1: slight, 2: moderate, 3: severe.

Samples were lactoferrin-positive in 49 patients and lactoferrin-negative in 29 patients (Table 2). Mean levels of fecal calprotectin concentration were 686 mg/kg (ranging from 52, 98 to 2542, 86 mg/Kg). The area under the ROC curve to analyze the cut-off was 0,939 (Figure 1). The best global cut-off was 200, 01 mg/Kg (sensitivity 88, 6; specificity 97, 1). Calprotectin was present at levels above the 200 mg/kg in 40 patients whose biopsies showed intestinal inflammation (Table 3). As the fecal calprotectin test was quantitative, we chose to evaluate all levels of fecal calprotectin found and to compare them to the degree of inflammation as defined by the histological study of the patients as a whole (Figure 2). All data were analyzed with respect to sensitivity, specificity, positive predictive value, negative predictive value, and accuracy when compared to endoscopic (Table 4) and histological evaluation (Table 5).

**Table 2:** Comparison between fecal lactoferrin assessment and histological evaluation.

	Histological evaluation				
		
Fecal lactoferrin	Crohn's Disease with Inflammation	Ulcerative Colitis with Inflammation	Crohn's Disease without Inflammation	Ulcerative Colitis without Inflammation	Total
**Positive**	22 (92%)	25 (89.3%)	2 (14.3%)	0 (0%)	49(62.8%)
**Negative**	2 (8%)	3 (10.7%)	12 (85.7%)	12 (100%)	29(37.2%)
**Total**	24 (100%)	28 (100%)	14 (100%)	12 (100%)	78 (100%)

p = 0.000 (Spearman rank correlation)

**Table 3:** Comparison between fecal calprotectin and histological evaluation.

	Histological evaluation				
		
Fecal calprotectin	Crohn's Disease with Inflammation	Ulcerative Colitis with Inflammation	Crohn's Disease without Inflammation	Ulcerative Colitis without Inflammation	Total
**Positive (>200 ng/ml)**	20 (83.3%)	20 (71.4%)	0 (0%)	0 (0%)	40(51.3%)
**Negative**	4 (16.7%)	8 (28.6%)	14 (100%)	12 (100%)	38 (48.7%)
**Total**	24 (100%)	28 (100%)	14 (100%)	12 (100%)	78 (100%)

p = 0.000 (Spearman rank correlation)

**Table 4:** Statistical analysis of activity indexes, blood tests, and fecal markers.

	Sensitivity	Specificity	PPV	NPV	Accuracy
**CDAI**	26,1%	100%	100%	46,9%	55,3%
**CRP**	63,6%	73,5%	75,7%	61%	67,9%
**Lactoferrin**	93,2%	76,5%	83,7%	89,7%	85,9%
**Calprotectin**	88,6%	97,1%	97,5%	86,8%	92,3%

**Table 5:** Statistical analysis of activity indexes, blood tests, and fecal markers.

	Sensitivity	Specificity	PPV	NPV	Accuracy
**CDAI**	25%	100%	100%	44%	53%
**CDEIS**	92%	93%	96%	87%	92%
**MDAI**	75%	100%	100%	63%	83%
**CRP**	58%	73%	81%	46%	63%
**Lactoferrin**	90%	92%	96%	83%	91%
**Calprotectin**	77%	100%	100%	68%	85%

### Correlation between fecal markers, clinical activity index (CDAI), endoscopy (CDEIS) and clinical-endoscopy (MDAI)

In patients with CD, lactoferrina and calprotectin results were found to correlate with CDAI (p = 0,043; p = 0,010, respectively) and the CDEIS results (p = 0.000; p = 0.000, respectively). In patients with UC, lactoferrina and calprotectin correlated with MDAI results (p = 0.000; p = 0,000; respectively).

### Correlation between fecal markers and blood tests (C-reactive protein)

Lactoferrin and calprotectin findings correlated only with C-reactive protein findings in both the CD and UC groups (p = 0.006; p = 0.000).

## Discussion

Inflammation is the basis for many signs and symptoms of disease, making its detection and monitoring fundamental to clinical management. At present, a majority of studies have shown that the best way to assess inflammation is through endoscopic and/or histological evaluation [[1,8], and [9]]. Patients' symptoms can be indicators of inflammation and disease activity, but these are subjective and are often influenced by disease factors that are not inflammatory (fibrosis). Various clinical indices developed to calculate inflammatory activity among patients with IBD rely on the combination of signs and symptoms [1].

One means to assess inflammation that has been discussed in recent years is the analysis of the infiltration of neutrophil in the intestinal mucosa and their transmigration to the lumen [10]. When intestinal inflammation occurs, fecal lactoferrin rises rapidly and correlates with endoscopic and histological alterations in patients with IBD, supporting the idea that it is a sensitive and specific means to identity inflammatory activity in these patients [7].

Several authors working on the role of fecal lactoferrin in IBD patients have shown that concentrations of lactoferrin are significantly higher in patients with active disease than in those with inactive disease [7]. These results are consistent with those found in the current study, in which we found that samples from 49 out of 52 patients with intestinal inflammation detected by histological evaluation were positive for lactoferrin.

Another marker derived from neutrophil that has shown great promise for identifying intestinal inflammation is fecal calprotectin [1,11-17]. In patients with active IBD, calprotectin values vary between 200 mg/kg and 20,000 mg/kg [4,18-21].

Many authors have claimed that calprotectin levels correlate closely with histological evaluation than macroscopic findings, suggesting that this biological marker is more sensible than endoscopy in evaluating IBDs activity [4,8]. Furthermore fecal calprotectin concentrations predicted the severity of colorectal inflammation, with advanced histological grades of colorectal inflammation [4]. In our study has demonstrated that more intense levels of inflammation are associated with elevated calprotectin values, demonstrating a significant correlation between calprotectin and the severity of inflammation. However the accuracy of calprotectin predicted inflammation when compared with endoscopic and histological were 92, 3% and 85%.

Calprotectin determination appears to better reflect disease activity in UC than CD [21,22]. As an example, Costa et al. found that fecal calprotectin levels above 50 ug/g were better correlated with the colitis activity index than the CDAI [21]. The relatively poor correlation between calprotectin levels and CDAI might indeed not be due to a calprotectin pitfall, but to the fact that CDAI is mostly a clinical score and is not sensitive enough to detect subclinical activity of the disease, which is known to occur rather frequently in CD. Nevertheless, several studies could not demonstrate a correlation between fecal calprotectin and clinical activity of CD (evaluated by CDAI) [23,24] or endoscopic lesions (evaluated by CDEIS) [24].

Sipponen et al. found that both fecal Calprotectin and lactoferrin correlated significantly with CDEIS (Spearman's r 0,729, p < 0,001). With a cut-off level of 200 microg/g for a raised fecal Calprotectin concentration, sensitivity was 70%, specificity 92% in predicting endoscopically active disease [25].

Other studies have not been able to demonstrate correlation between fecal calprotectin concentration and UCs clinical activity [6]. It has been suggested, but not proved; that CD patients' stratification based on phenotypical pattern (inflammatory, stricturing or fistulizing) could improve calprotectin's predictive capacity for this disease. As calprotectin is an inflammation marker, its predictive role will probably produce best results in the inflammatory pattern of the disease [26]. In summary, the exact strength of any correlation of fecal calprotectin levels with disease activity indicators is therefore not well established at present [27].

In patients with Colitis, it was observed that all patients with clinical and endoscopic signs of inflammation (as determined by the MDAI) had lactoferrin and calprotectin presents in their stool. These findings also occurred in patients with CD; however there were few patients with CDAI higher than 150 points, so further studies are required to resolve this issue.

Both lactoferrin and calprotectin were present in the majority of samples from patients with elevated CRP, but we didn't find a good correlation with fecal markers and others laboratory parameters. Hemoglobin, hematocrit, leukocytes, platelets and ESR are routinely used as inflammatory markers in blood when IBD is suspected. However, these markers correlate poorly with endoscopic and histological [9], as we also found.

## Conclusion

Several conclusions can be made from the present results. Firstly, fecal lactoferrin and calprotectin are sensitive and specific markers for the detection of intestinal inflammation in IBD patients. Secondly, fecal calprotectin levels are directly proportional to the degree of inflammation in the intestinal mucosa.

## Abbreviations

CDAI: Crohn's Disease Activity Index; MDAI: Mayo Disease ActivityIndex; CDEIS: Crohn's Disease Endoscopic Index of Severity.

## Competing interests

The authors declare that they have no competing interests.

## Authors' contributions

AV performed all manuscript; CBF contributed to manuscript; EGR helped to draft the text; WAK helped to draft the text; LGBR did double-balloon enteroscopy and contributed to manuscript; PAC did colonoscopy and contributed to manuscript. All authors read and approved the final manuscript.
